# A comparative study of post-warming survival rates and clinical outcomes of human blastocysts vitrified/warmed by CryoTouch and Cryotop methods

**DOI:** 10.5935/1518-0557.20210116

**Published:** 2022

**Authors:** Somayeh Keshavarzi, Azadeh Dokht Eftekhari, Hajar Vahabzadeh, Marzieh Mehrafza, Robabeh Taheripanah, Masoumeh Asgharnia, Sahar Esfandyari, Alaleh Ghazifard, Hossein Hosseinirad, Shahrokh Paktinat

**Affiliations:** 1 Infertility Clinic, Erfan Niayesh Hospital, Iran University of Medical Sciences, Tehran, Iran; 2 Mehr Fertility Research Center, Guilan University of Medical Sciences, Rasht, Iran; 3 Men's Health and Reproductive Health Research Center, Shahid Beheshti University of Medical Sciences, Tehran, Iran; 4 Islamic Azad University, Rasht Branch, Rasht, Iran; 5 College of Medicine, University of Illinois at Chicago, Chicago, IL, USA

**Keywords:** cryopreservation, embryo vitrification, survival rate, clinical outcome

## Abstract

**Objective:**

Advances in embryo culture conditions and the development of vitrification as a revolutionary cryopreservation method have allowed for routine use of blastocyst transfer in assisted reproduction technology (ART) cycles. Several vitrification/warming media and devices have been introduced for commercial use so far. The aim of this retrospective study was to compare post-warming survival rates and clinical outcomes of human blastocysts vitrified/warmed by two different commercial methods (CryoTouch and Cryotop) during ART cycles.

**Methods:**

This retrospective study assessed a total of 50 frozen embryo transfer (FET) cycles conducted on 56 warmed blastocysts between January 2018 and December 2020. Post-warming blastocyst survival rates and clinical outcomes including clinical pregnancy and live birth rates were calculated after single blastocyst transfer cycles.

**Results:**

The results revealed no significant differences between two groups in post-warming survival rate (*p*-value=0.8381), clinical pregnancy rate (*p*-value=0.8157) and live birth rate (*p*-value=0.7041).

**Conclusions:**

Post-warming survival rates and clinical outcomes were comparable with no significant difference in blastocysts vitrified/warmed by CryoTouch and Cryotop commercial methods.

## INTRODUCTION

Successful clinical pregnancies with cryopreserved embryos were first reported in early 1980s (Al-Azawi *et al*., 2013). Ever since, cryopreservation has become a routine method in infertility clinics as it gives the opportunity to transfer the frozen/warmed embryos to uterus whenever the endometrium is optimally prepared. Over the past decades, the techniques of embryo cryopreservation have evolved to heighten the post-warming survival rate and clinical outcomes (Al-Azawi *et al*., 2013). Currently, vitrification and slow freezing are the available methods for embryo cryopreservation (Serdarogullari *et al*., 2019). During vitrification, ice crystal formation and growth are avoided by ultrafast freezing of the embryonic cells using a simple procedure without needing any costly device (Serdarogullari *et al*., 2019), while slow freezing requires expensive equipment and more time. Due to the ease of use, rapid procedure and low cost of vitrification, it has become the method of choice worldwide and nearly all of the clinics utilize this method for cryopreservation of embryos in any developmental stage (Fasano *et al*., 2014; Wirleitner *et al*., 2016), and there are multiple reports of improved post-warming embryo survival rate and clinical outcomes using vitrification for human cleavage and blastocyst stage embryos in comparison to slow freezing (Cobo *et al*., 2012).

As superior clinical results of blastocyst transfer over cleavage-stage embryo transfer have been revealed by a number of investigators (Wang *et al*., 2021), there is an increasing trend towards extended culture of embryos until blastocyst stage. Nowadays, the single blastocyst transfer strategy has become popular based on improved clinical results (Wang *et al*., 2021). After transferring the selected blastocyst to the uterus, surplus blastocysts must be vitrified for later use. The blastocyst is sensitive to ice crystal formation during vitrification as the blastocoele cavity contains a large amount of water. Therefore, blastocyst vitrification must be performed carefully to reduce the risk of ice crystal formation.

The quality of blastocysts, duration of exposure to cryoprotectants and volume of the solution used for loading the embryos on the vitrification device largely affect the survival rate after warming and the subsequent clinical results (Zeng *et al*., 2018). In addition, the composition of vitrification/warming media (solutions) and vitrification device have massive effects on the final results. Today, the use of vitrification/warming media and device from the same manufacturer is more common and investigators tend to use the term "vitrification method" when performing vitrification/warming procedures using products of a single company. This nomenclature is often based on the name of vitrification device, for instance Cryotop method which is introduced by Kitazato BioPharma Co. (Cobo *et al*., 2008; 2012; Kuwayama, 2007).

Previous reports have mostly focused on recognizing the best cryopreservation method with the maximum clinical yields (Serdarogullari *et al*., 2019; Fasano *et al*., 2014; Wirleitner *et al*., 2016). To the best of our knowledge, very little to no effort has been made to compare different vitrification methods in terms of final results despite the wide variety of the commercial products. It is expected that various commercial products have different pros and cons. In addition, the availability of the product for assisted reproductive technology (ART) clinic may also influence the clinical results due to the instability of the media components.

This retrospective study aimed at assessment of the post-warming survival rate and clinical outcomes of blastocyst stage embryos vitrified/warmed using the commercial products introduced by Ravan Sazeh Co. (CryoTouch method) and compare them with the products of Kitazato (Cryotop method).

## MATERIALS AND METHODS

### Study population

Infertile couples referred to Erfan Niayesh Hospital Tehran, Iran and Mehr Fertility Research Center, Rasht, Iran, between January 2018 and December 2020 were evaluated for eligibility to enter this retrospective study. Institutional Review Board of each center approved the study protocol based on the Ethical Principles for Medical Research.

Registered documents of each couple were reviewed and information regarding demographic characteristics, ovarian stimulation protocol, fertilization method, embryo culture technique, embryo grading system, vitrification/warming protocols, frozen embryo transfer (FET) protocol, endometrial preparation method and clinical outcomes were all extracted. A total of 50 FET cycles using blastocysts vitrified/warmed on day 5 of development were considered to enter the study after consideration of the following inclusion/exclusion criteria:

-The inclusion criteria consisted of age range between 20-38 years old, body mass index (BMI)<30 kg/m^2^, serum level of follicle-stimulating hormone (FSH)<10 mIU/ml on the day 3 of menstrual cycle, having more than 5 blastocyst stage embryos produced by intracytoplasmic sperm injection (ICSI) fertilization method and undergoing their first ICSI/FET cycle with gonadotropin-releasing hormone (GnRH) antagonist protocol for ovarian stimulation.

-The exclusion criteria were presence of space-occupying lesions in the uterus, anatomical anomalies, hormonal dysfunctions, ovarian hyperstimulation syndrome (OHSS), immunological diseases and syndromes, hydrosalpinx, endometriosis, ectopic pregnancy, repeated implantation failure (RIF), miscarriage, and having less than 5 blastocyst stage embryos produced by ICSI method.

### Ovarian stimulation protocol

All of the selected couples had received GnRH antagonist protocol for ovarian stimulation. The protocol was initiated by the administration of estradiol valerate (2 mg, PO, BID; Aburaihan Co., Tehran, Iran) from day 21 of the previous cycle and continued until days 2-4 of the subsequent cycle. Afterwards, recombinant FSH (150-225 IU, daily; Gonal F, Merck, Germany) was administered from day 2 or 3 of the cycle. Transvaginal ultrasonography was performed to monitor the follicular growth. The administration of GnRH antagonist (Cetrotide, Merck, Germany) was performed when at least one follicle reached the size of 13-14 mm in diameter and the administration continued until the day of ovulation induction. The final oocyte maturation and ovulation induction was conducted by injection of human chorionic gonadotropin (hCG, 10,000 IU; Choriomon, IBSA, Switzerland). Finally, ovum pick-up (OPU) was performed 36 hours after hCG injection.

### Cumulus cell-oocyte complex retrieval, oocyte denudation and fertilization

Culture media preparation was performed 8 hours prior to use by adding protein supplement (10% V/V of human serum albumin) and equilibration at 37°C in 6% CO_2_ incubator. Cumulus cell-oocyte complexes (COCs) were retrieved from follicular fluid immediately after OPU. Then, the COCs were washed in a handling medium (HTF w/HEPES, Fertilite^®^, Ravan Sazeh Co., Tehran, Iran) and maintained in embryo culture medium (SingleCulture Medium, Fertilite^®^, Ravan Sazeh Co., Tehran, Iran) until denudation. Oocyte denudation was performed using both enzymatic (Hyaluronidase, Fertilite^®^, Ravan Sazeh Co., Tehran, Iran) and mechanical methods. ICSI was conducted on all metaphase II oocytes 3-4 hours after OPU. Afterwards, the injected oocytes were cultured in 30-50 µL of first step sequential embryo culture medium (G1 Medium, Fertilite^®^, Ravan Sazeh Co., Tehran, Iran) under mineral oil (RS Medical, Ravan Sazeh Co., Tehran, Iran) overlay. After 3 days, the embryos were transferred to fresh second step sequential embryo culture medium (G2 Medium, Fertilite^®^, Ravan Sazeh Co., Tehran, Iran) and cultured until blastocyst stage. The resulting blastocysts were evaluated morphologically and top-quality blastocysts were selected for vitrification.

### Blastocyst quality assessment

Blastocyst grading was performed according to the Gardner grading system (Wirleitner *et al*., 2016) as following: expansion score 0 = no cavity, score 1 = blastocoel cavity less than half volume of the embryo, score 2 = blastocoel cavity more than half volume of the embryo, score 3 = blastocoel cavity completely filling the embryo, score 4 = blastocoel cavity larger than the embryo and thinning zona, score 5 = hatching blastocyst; for inner cell mass (ICM), Grade A = formed by many tightly packed cells, Grade B = several loosely packed cells, Grade C = few cells; for trophectoderm (TE), Grade A = many cells forming a cohesive layer, Grade B = few cells and loose layer, Grade C = very few large cells. Top-quality blastocysts were defined as blastocysts with expansion grades 4-5 or 2-3, and ICM and TE with AA, AB or BA classifications.

### Vitrification and warming procedures

Top-quality blastocysts were selected for the vitrification using two commercial methods and the procedure was conducted according to the manufacturer's protocol, CryoTouch method (RS Medical, Ravan Sazeh Co., Tehran, Iran) or Cryotop method (Kitazato BioPharma Co., Shizuoka, Japan). Both protocols were similar with little difference. In brief, the equilibration was conducted in equilibration solution for 12-15 minutes in both CryoTouch and Cryotop methods at room temperature. Then, the blastocysts were placed in vitrification solution, then aspirated with minimum volume of the vitrification solution and finally placed onto the tip of CryoTouch^®^ or Cryotop^®^ vitrification devices within 50-60 sec. The loaded devices were immediately put vertically into liquid nitrogen, then placed in goblet and stored in liquid nitrogen tank.

In the morning of blastocyst transfer day, the embryos were warmed in the corresponding commercial media using the protocol provided by each manufacturer, CryoTouch and Cryotop methods. Briefly, the vitrification device was rapidly removed from liquid nitrogen and immersed in prewarmed warming solution. Then, the blastocysts were detected and immediately transferred to another droplet of warming solution and incubated for 1 minute. Subsequently, the blastocysts were incubated in the dilution solution for 3 minutes. Next, the blastocysts were incubated in the washing solution for 5 minutes. Eventually, the blastocysts were maintained in embryo culture medium (G2 Medium, Fertilite^®^, Ravan Sazeh Co., Tehran, Iran) until FET.

### Endometrial preparation and embryo transfer

Hormone replacement therapy (HRT) was conducted for endometrial preparation. First, estradiol valerate (6 mg/day, PO; Aburaihan Co., Tehran, Iran) was initiated from day 2-3 of the menstrual cycle and continued up to 8 mg/day until the endometrial thickness reached 8 millimeters. Then, progesterone (400 mg, suppository, BID; Cyclogest, Actavis, England, UK) was administered when the endometrial thickness was upper 8 millimeters. In the presence of positive result for β-hCG test, the estradiol and progesterone administrations were continued until weeks 6 and 12 of gestation, respectively.

Embryo transfer (ET) was performed by an expert gynecologist using an embryo transfer catheter (Guardia(tm) Access, Cook, USA) under the ultrasonography guide according to the guideline provided by American Society for Reproductive Medicine (ASRM). Single top-quality vitrified/warmed blastocyst was selected for each ET cycle in each couple.

### Outcome assessment

The post-warming blastocyst survival rate was calculated as the percentage of survived blastocysts after warming. Clinical pregnancy rate was calculated from the number of observed gestational sacs by ultrasonography per blastocyst transfer. Live birth rate was calculated from the number of live births per the blastocyst transfer.

### Statistical analysis

All the obtained data were analyzed using GraphPad Prism software (V8, US). Comparisons of the means were conducted by Student's t test. The *p*-value was considered significant at < 0.05 level. Data are represented as mean ± standard deviation (SD).

## RESULTS

A total of 50 ICSI/FET cycles were evaluated during the study. Among these cycles, 27 vitrification/warming cycles were performed using CryoTouch method and 23 vitrification/warming cycles were conducted by Cryotop method. As shown in Table 1, there were no significant difference between the two groups in mean age, BMI, duration of infertility, type of infertility (primary or secondary), serum level of day 3 FSH, serum level of Anti-Mullerian hormone (AMH), and sperm count of spouse.

**Table 1.** Demographic features of the studied groups.

Characteristics	Groups		
	Cryotop method	CryoTouch method	*p*-value
Number of ICSI/FET cycles	23	27	-
Age (years)	33.04±4.61	31.15±5.41	0.1933
BMI	26.00±1.97	26.89±2.27	0.1505
Duration of infertility (years)	5.82±2.34	4.33±2.86	0.0523
Type of infertility (primary)	78.26%	66.67%	0.3731
Type of infertility (secondary)	21.74%	33.33%	0.3731
FSH (Day 3) (mIU/ml)	5.37±1.32	4.66±1.55	0.0895
AMH (ng/ml)	3.59±1.56	3.82±2.42	0.6916
Sperm (count/ml)	(58±50) ×106	(75±53) × 106	0.2393

AMH: Anti-Mullerian hormone; BMI: Body mass index; FSH: Follicle stimulating hormone; NS: Non-significant.

Table 2 represents the characteristics of the studied patients during ovarian stimulation including total dose and duration of gonadotropin, serum levels of luteinizing hormone (LH), Estradiol (E2) and Progesterone (P) on trigger day, number of retrieved COCs, and percent of top-quality blastocysts resulted from ICSI. According to the results, there were no significant difference in these characteristics between the patients who were undergone ICSI/FET cycles using the CryoTouch or Cryotop methods.

**Table 2.** Ovarian stimulation characteristics and outcomes in the studied groups.

Characteristics	Groups		
	Cryotop method	CryoTouch Method	*p*-value
Total dose of administrated gonadotropin (IU)	1843±321	1907.57±445	0.5635
Duration of gonadotropin administration (days)	10.74±1.28	10.81±0.92	0.8101
LH (IU/mL) on day of trigger	5.13±7.64	4.17±0.86	0.5208
E2 (pg/mL) on day of trigger	2923±704	2748±590	0.3445
P (IU/mL) on day of trigger	0.72±0.31	0.89±0.29	0.0548
Number of retrieved COCs	14.6±4.3	11.2±5.2	0.1898
Number of top-quality blastocysts	2.60±1.07	3.40±1.86	0.0764

A total of 56 blastocysts were warmed and 50 ET cycles were conducted using single top-quality blastocyst. Among 56 blastocysts, 30 were vitrified/warmed using CryoTouch method and 26 were vitrified/warmed using Cryotop method.

Post-warming survival rates of blastocysts were 94.44% for CryoTouch method (95% CI 88.11-100.8) and 93.48% for Cryotop method (95% CI 86.03-100.9), with no significant difference between the groups (*p*-value=0.8381) (Table 3).

**Table 3.** Post-warming survival rate and clinical outcomes of vitrified/warmed blastocysts in the studied groups.

Variables	Groups		
	Cryotop method (n=23)	CryoTouch Method (n=27)	*p*-value
Number of warmed blastocysts	26	30	-
Blastocyst post-warming survival rate	93.48%	94.44%	0.8381
Clinical pregnancy rate	47.83%	44.44%	0.8157
Live birth rate	34.78%	29.63%	0.7041

Clinical pregnancy rates were 44.44% for CryoTouch method (95% CI 24.41-64.48) and 47.83% for Cryotop method (95% CI 25.74-69.91), with no significant difference between the groups (*p*-value=0.8157) (Table 3).

Live birth rates were 29.63% for CryoTouch method (95% CI 11.22- 48.04) and 34.78% for Cryotop method (95% CI 13.72-55.84), with no significant difference between the groups (*p*-value=0.7041) (Table 3).

## DISCUSSION

Herein, the results revealed no difference in clinical outcomes of blastocysts that were vitrified/warmed by RS CryoTouch or Cryotop methods. Indeed, post-warming survival rate, clinical pregnancy and live birth were comparable in both commercial products.

Embryo development to the blastocyst stage requires successful genomic activation and passing critical developmental steps, therefore when an embryo reaches this stage it is considered to be highly competent (Youssry *et al*., 2008). In the past decade, single blastocyst transfer has become a common procedure in ART clinics worldwide because of more success rates in clinical results (Wang *et al*., 2021). This necessitates a reliable cryopreservation method for saving the remaining blastocysts which are to be used in the following transfer cycles.

There are multiple variables which impact the survival rates of the embryos during vitrification/warming processes. The most important factors that influence the effectiveness of the procedure include the concentration of cryoprotectants in vitrification/warming solutions, duration of embryo exposure to these cryoprotectants, temperature in which the procedure is being performed and the type of vitrification device which influences the cooling rate and the size of vapor coat (Al-Azawi *et al*., 2013). An ideal strategy for improving the efficiency of vitrification is to increase the speed of thermal conduction and decrease the concentration of cryoprotectants. As most of the cryoprotectants included in commercial vitrification solutions have some degrees of cellular toxicity, it is always desired to formulate a solution containing a mixture of cryoprotectants with the lowest toxicity for embryonic cells (Mori *et al*., 2015). As claimed by the manufacturers, both CryoTouch and Cryotop methods' vitrification media contain similar cryoprotectants with comparable concentrations (Mori *et al*., 2015; Mori & Kuwayama, 2009). Therefore, the observed similarity in blastocyst survival rate after vitrification/warming using these commercial methods may be the consequence of this similarity of components and indicates their equality in efficiency for human blastocyst vitrification.

It is assumed that prolonged exposure to cryoprotectants may increase the possibility of toxicity (AbdelHafez *et al*., 2010; Loutradi *et al*., 2008). It is always desirable to enhance the speed of the vitrification procedure in order to avoid possible toxicities. In addition, high speed will reduce the likelihood of osmotic injury as the embryos are held in solutions without mineral oil overlay during vitrification/warming procedures (Fasano *et al*., 2014). The incubation time in the equilibration solution was equal in both CryoTouch and Cryotop methods.

Different protocols have been presented for vitrification so far. Due to the differences in basic characteristic of the studied population as well as technical differences in ART clinics, the reported clinical outcomes are not always homogeneous (Wirleitner *et al*., 2016; De Vos *et al*., 2016; Debrock *et al*., 2015). In a study conducted on 6019 embryos undergoing vitrification/warming using Cryotop method in different developmental stages, it was concluded that nearly 95% of day 5 blastocysts had 100% of intact cells (Cobo *et al*., 2012). A survival rate of 95% was also reported by Ferreux *et al*. (2018) in a retrospective cohort follow-up study conducted on 1347 frozen-thawed blastocysts. In line with these studies, we showed ~94% blastocyst survival rate using both CryoTouch and Cryotop methods. The efficiency of Cryotop method for vitrification/warming of human embryo and oocyte is demonstrated in previous studies (Cobo *et al*., 2012; Braga *et al*., 2016; Liu *et al*., 2020; La Marca *et al*., 2019). However, there is no published data, to the best of our knowledge, regarding the efficiency of CryoTouch method for blastocyst cryopreservation despite its routine use in infertility clinics of Iran.

Over the past decade, the number of reported live births obtained from vitrified/warmed blastocysts has substantially increased (Al-Azawi *et al*., 2013; Wang *et al*., 2021). However, the reported data for clinical pregnancy and live births are not homogenous. Cobo *et al*. (2012) reported pregnancy rate of 43%, and live birth rate of 40.6% with vitrified/warmed blastocysts. Kaye *et al*. (2017) reported a clinical pregnancy rate of nearly 63% after single vitrified/warmed blastocysts transfer. Ferreux *et al*. (2018) reported a clinical pregnancy rate of 43.2% and live birth rate of 29.6% resulting from vitrified/warmed blastocysts. Our results for clinical pregnancy and live birth rates were comparable with these mentioned studies. Furthermore, we showed no difference in clinical pregnancy and live birth rates between blastocysts vitrified/warmed using CryoTouch and Cryotop methods.

Although successful pregnancies are reachable following the transfer of embryos with less than 50% of the survived cells post-warming (Veiga *et al*., 1987), pregnancy rates are higher when all the embryonic cells survive during vitrification/warming procedure. Indeed, if embryos survive the process with all its cells, the pregnancy rate will be comparable with that of fresh ET cycles (Al-Azawi *et al*., 2013). Human embryo at blastocyst stage has different physiological requirements than cleavage stage embryo. These differences influence the chance of survival after being subjected to non-physiological conditions like vitrification (Zeng *et al*., 2018). A major factor that has effect on survival rate of the blastocyst is the blastocoele cavity. As expected, ice crystal formation is directly proportional to the volume of the cavity (Ochota *et al*., 2017). Recently, artificial shrinkage of the blastocoele cavity is proposed to enhance the post-warming survival rate of expanded blastocysts (Darwish & Magdi, 2016; Kovačič *et al*., 2018). Overall, it is essential to consider these differences of blastocyst and cleavage stage embryos in efforts to establish a simple and reliable procedure to optimize blastocyst vitrification in order to have the best possible clinical outcome.

## CONCLUSIONS

In conclusion, similar outcomes were observed in blastocysts vitrified/warmed using CryoTouch and Cryotop commercial methods. These results provide strong evidence regarding the comparable efficiency of vitrification/warming media and device produced by Ravan Sazeh Co. (CryoTouch method) and Kitazato (Cryotop method). This is hopeful for infertility clinics in Iran that are routinely using CryoTouch method because of its availability, low cost, and prolonged expiration date as it is domestically manufactured in Iran.

## Abbreviations

AMH: Anti-Mullerian hormone
ART: Assisted reproductive technology
ASRM: American Society for Reproductive Medicine
BMI: Body mass index
COCs: Cumulus cell-oocyte complexes
E2: Estradiol
ET: Embryo transfer
FET: Frozen embryo transfer
FSH: Follicle-stimulating hormone
GnRH: Gonadotropin-releasing hormone
hCG: Human chorionic gonadotropin
HRT: Hormone replacement therapy
ICSI: Intracytoplasmic sperm injection
IVF: In vitro fertilization
LH: Luteinizing hormone
OHSS: Ovarian hyperstimulation syndrome
OPU: Ovum pick-up
P: Progesterone
RIF: Repeated implantation failure
